# Introducing CRIS—A unified framework for systematic literature searches across disciplines

**DOI:** 10.3389/fpubh.2025.1489161

**Published:** 2025-06-13

**Authors:** Celina Ciemer, Thomas Jürgen Klotzbier, Sabiha Ghellal, Nadja Schott

**Affiliations:** ^1^Institute for Sport and Movement Science, University of Stuttgart, Stuttgart, Germany; ^2^Institute for Games, Stuttgart Media University, Stuttgart, Germany

**Keywords:** cross-disciplinary, interdisciplinary, systematic review, literature search, user experience, game design, human movement science

## Abstract

**Introduction:**

Cross-disciplinary research approaches have become more necessary in light of the increasing global and societal challenges. As different forms of collaboration emerge, cross-disciplinary systematic reviews that integrate these approaches are increasingly recognized as crucial. A key part of these reviews is conducting a valid and thorough literature search. To comprehend the state of knowledge, integrating diverse findings and ensuring that the literature search captures relevant studies from all viewpoints, including their combinations and collaborations, is important.

**Objective:**

This article presents a framework for conducting cross-disciplinary literature searches that adhere to established best-practice guidelines and reporting standards. The framework seeks to include research across all forms of collaboration across disciplines, including multidisciplinary, interdisciplinary, and transdisciplinary. The objective is to enhance the sensitivity and robustness of literature searches in cross-disciplinary research contexts.

**Methods:**

We developed a framework that integrates a pre-process into the search to support cross-disciplinary literature searches. Additionally, we derived a procedure from specific concepts, including the use of shared thesaurus, focus, and iterative approach, which are applied throughout the various stages of the process. To demonstrate the value of the cross-disciplinary literature search (CRIS) framework, we performed an example search that combines User Experience and Game Design with Human Movement Science. We conducted three literature searches and compared our framework with discipline-specific and an expert overlap perspectives.

**Results:**

By applying our CRIS framework, we observed significant improvements in sensitivity and robustness compared to the other searches, illustrating the framework's effectiveness in cross-disciplinary research settings.

**Conclusion:**

Through our example, which combines User Experience and Game Design with Human Movement Science, we show that our framework substantially enhances the quality of literature searches, underscoring its potential for advancing cross-disciplinary research.

## 1 Introduction

In today's world, the growing complexity of global and societal challenges has brought cross-disciplinary research to the forefront (1). Transcending single disciplines, cross-disciplinary research addresses topics such as global crises and specific human life issues such as environmental degradation and health care (1–5). The National Academies of Sciences, the National Academy of Engineering, and the Institute of Health (6) highlight the role of collaboration in research in addressing complex problems, mainly through the potential of emerging technologies to transform existing disciplines and create new fields of study or domains. It is not limited to hybrid disciplines but also includes collaborations across disciplines that are brought together specifically for a particular topic (7). In this article, the term cross-disciplinary collaboration is used according to Rosenfield (8) and Aagaard-Hansen (9) as an umbrella term encompassing all forms of collaboration. These forms can differ in the processes used for problem-solving. The most common types are multidisciplinary, interdisciplinary, and transdisciplinary collaboration (10–15).

Systematic reviews are essential because they provide a comprehensive overview by synthesizing diverse research on complex topics. By systematically identifying and analyzing recent studies, they offer a deeper understanding of a specific subject (16, 17). There is a growing need to extend systematic reviews beyond their traditional disciplines, encouraging their application in fields that have not yet widely adopted this methodology (18, 19). However, from a cross-disciplinary perspective, systematic reviews play a crucial role in achieving a comprehensive understanding of a topic by integrating different disciplines. By identifying knowledge gaps beyond the expertise of a single discipline, they contribute to a more open and unified perspective (20–22).

The comprehensiveness of a systematic review depends heavily on how the literature search is conducted. This includes a methodological approach that ensures comprehensive coverage, accuracy, impartiality, reproducibility, and relevance. The strength and reliability of a review's findings are directly related to the quality of this search process (17, 23, 24).

However, systematic reviews introduce new challenges when viewed from a cross-disciplinary perspective. Different disciplines often follow different research approaches, including variations in goals, methodologies, terminologies, and standards, and may also exhibit epidemiological differences, which can make a unified view difficult (9). In particular, cross-disciplinary systematic reviews face the unique challenge of covering a broad scope to ensure that no aspect of a cross-disciplinary topic is overlooked while simultaneously managing the evolving nature of specialized language, integrating diverse perspectives from different articles, and balancing the varying emphases of various disciplines within a single systematic review (20, 21, 25). This requires new strategies of literature search tailored to cross-disciplinary needs (20). Therefore, it is crucial to approach a systematic review from a cross-disciplinary perspective from the very beginning.

This article provides a framework for conducting a CRoss-dIsciplinary literature Search (CRIS) in the context of systematic reviews. We aim to develop a framework that includes a procedure and methods tailored to the specific needs of open and unified questions in cross-disciplinary systematic reviews. To this end, we draw on foundations from Human-Computer Interaction (HCI) and Design Thinking (DS) research, which focus on fostering collaboration and providing problem-solving methods (26–28).

We demonstrate our framework with an example of a cross-disciplinary systematic review that combines User Experience and Game Design (UXG) and Human Movement Science (HMS), specifically focusing on “digital interactive experience- and game-based fall interventions for community-dwelling healthy older adults” (29). The chosen topic aligns with our team's expertise. The example validates our framework using relative sensitivity and overall robustness compared to discipline-specific and expert overlap searches.

## 2 Methods

### 2.1 Methods and guidance for framework development

In developing our framework, we adhere to the reporting standards outlined in the Preferred Reporting Items for Systematic Reviews and Meta-Analyses (PRISMA) guidelines (16, 30), including the PRISMA-S extension (31), which is specifically tailored to literature searches. Additionally, our framework is based on the recommendations outlined in the Cochrane Handbook for Systematic Reviews of Interventions (24, 32, 33). Within these guidelines, using the PICO structure (34) is widely recommended for defining the scope of a systematic review. PICO organizes a research question into four key elements: Patient/Problem (P), Intervention (I), Comparison (C), and Outcome (O). Although initially developed for medical research, it is mainly used for clinical questions in intervention studies. For complex topics or when interventions are not well-defined, the Cochrane Guidelines (24) advises that tailored approaches may be necessary, allowing for deviations from the traditional PICO structure.

For conducting literature searches, also tailored methods exist for complex topics. For example, the Palliative cAre Literature rEview iTeraTive mEthod (PALETTE) (35) is particularly useful for literature searches in palliative care due to its comprehensive and iterative approach, as recommended by Cochrane (24). It addresses the unique challenges of the field, such as the diversity of practices, the exploratory nature of research questions, and the ambiguity in key terminology. The method is organized into four clear and coherent phases, structured within a decision tree: developing the review question, building the search strategy, validating this strategy, and performing the search. Its iterative process allows for continuous refinement and adaptation of the literature search (35).

In addition, complementary search techniques can significantly improve the thoroughness and relevance of searches, leading to a more comprehensive and well-substantiated search process. These techniques include *berry picking, golden bullets, pearl growing*, and *citation tracking*. Berry picking involves refining a search iteratively based on newly discovered information. Instead of retrieving a complete set of results at once, information is gathered in fragments (the “berries”). Techniques such as journal browsing and footnote chasing help identify relevant sources (36). Golden bullets refer to key articles that meet the inclusion criteria of a systematic review. They inform Boolean search strategies through feature extraction and help validate the search by ensuring they appear in the results (35). Pearl growing expands the search by identifying keywords and index terms from relevant articles, increasing the corpus of relevant literature (37, 38). Citation tracking finds relevant studies by analyzing references cited in an article (backward tracking) and later works that cite it (forward tracking), leveraging collective expert judgment (39, 40).

Fostering collaboration among disciplines, our framework draws on foundations from HCI and DT research. HCI is an inherently interdisciplinary field that provides concepts, procedures, and methods to understand and improve human-system interactions (27, 28), while DT offers a structured approach to solving complex problems (26). It combines empathy and a deep understanding of user needs as well as the problem's context with creativity in generating solutions, as well as a rational approach to their analysis and evaluation (41–44). Both HCI and DT actively engage experts from various disciplines, emphasizing methods and techniques that integrate diverse perspectives and expertise (45, 46). A concept used is the creation of a shared thesaurus, which establishes a common language and fosters empathy, allowing a deep understanding of the needs and requirements of each discipline (46–49). The context of the problem refers to the broader environment or situation in which it occurs or is considered (50, 51), including various external factors that can influence its perception, interpretation, and value. Incorporating context awareness can aid in understanding the complex construct within which the problem exists. Through an iterative process, solutions can be progressively optimized and tailored to meet the specific requirements of the disciplines involved and the context in which they operate (28). This can be achieved by alternating between creation and consumption parts (52).

### 2.2 Methods for framework evaluation

We compare CRIS with three common search strategies to assess its relative sensitivity and specificity. The evaluation follows principles of test validation. Search strategies 1 (HMS search) and 2 (UXG search) adopt a discipline-specific approach, where each discipline conducts searches independently based on its perspective without exchanging knowledge across disciplines. In contrast, search strategy 3 (HMS and UXG expert overlap search) relies on each discipline using its expert terminology, leading to a complete overlap of search results. The relative sensitivity was calculated by dividing the number of true positives identified using CRIS by the number of true positives found in each of the three common search strategies. While a precise specificity calculation was not feasible due to the lack of comprehensive data on the total number of incorrect entries across all information sources, we instead focused on the absolute number of entries that our framework filtered out.

## 3 Findings

### 3.1 The cross-disciplinary literature search framework

#### 3.1.1 Concepts and procedure

Cross-disciplinary systematic reviews face the unique challenge of incorporating diverse perspectives to ensure that no aspect of the topic is overlooked. These reviews must also navigate the evolving nature of specialized language while accommodating different articles' varying focuses and structures. Furthermore, it's crucial to integrate the distinct perspectives and priorities of the disciplines involved (20–22). We have developed specific concepts that form the foundation of our CRIS framework to address these challenges.

The *shared thesaurus* is a key concept developed to address the challenge of different languages used across disciplines. This concept aims to include the expertise of each discipline while fostering mutual understanding. The shared thesaurus incorporates both discipline-specific expert language and a more general language that represents an external view on the discipline. We refer to this as varying *specialized term depth*. This allows the search to capture results that reflect the terminology used in different forms of cross-disciplinary collaboration, as well as terms specific to a particular discipline, targeting specific academic audiences.

The key concept of *focus* addresses how different actors engage with a topic across various settings. It involves refining the focus within the context, which combines the perspectives of individual disciplines and the environments in which the research is situated. By positioning the research topic across diverse contexts, effective strategies can be developed to capture and address the subtleties of each setting. This includes understanding the intrinsic nature of the research topic, the disciplinary focus that examines how and why the topic is approached, and the database focus that emphasizes the clustering of related content.

The *iterative approach* is a key concept developed to address the challenge of integrating various disciplines throughout the process, ensuring that all perspectives are consistently represented while progressively moving toward common solutions. This concept is structured into two parts: Creation and Consumption. In the Creation part, the search strategy is designed or refined, while in the Consumption part, the strategy is evaluated, and insights are extracted. Iterative methods for systematic reviews are increasingly recommended as an effective way to address specific research questions (35, 53, 54).

**Figure 1:**
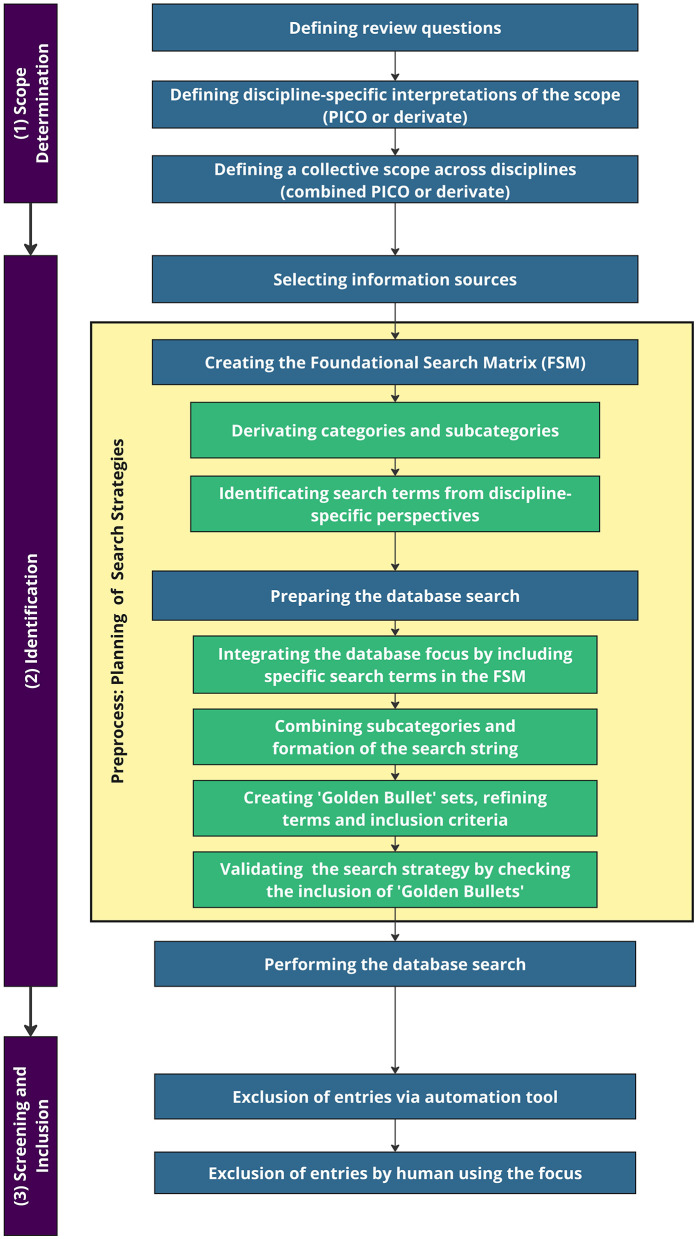
The procedure of the cross-disciplinary literature search (CRIS) framework for systematic reviews.

We developed a procedure to integrate these three key concepts into a unified search. This procedure aligns with recommended guidelines and includes a pre-process focused on the planning of search strategies. It is organized into three main phases: (1) Scope Determination, (2) Identification, and (3) Screening and Inclusion. Each phase is further divided into sub-phases, and results from one phase may prompt an iteration to a previous phase for refinement. A detailed description of the phases is presented in the walk-through and visualized in Figure 1.

#### 3.1.2 Definitions

In developing and evaluating our CRIS framework, we introduced additional terms. Basic concepts from set theory describe and quantify its components. The *search set* includes all database entries used in the search process, while the *solution set* comprises all results generated by the searches. This solution set is a union of various subsets, each representing a distinct search. The size of the solution set is largely influenced by the search strings used, which are crucial for capturing relevant results.


(1) $$\begin{array}{l} S=\overset{m}{\underset{n=1}{\wedge }}\left(S_{n}\right) \end{array}$$

**Figure 2:**
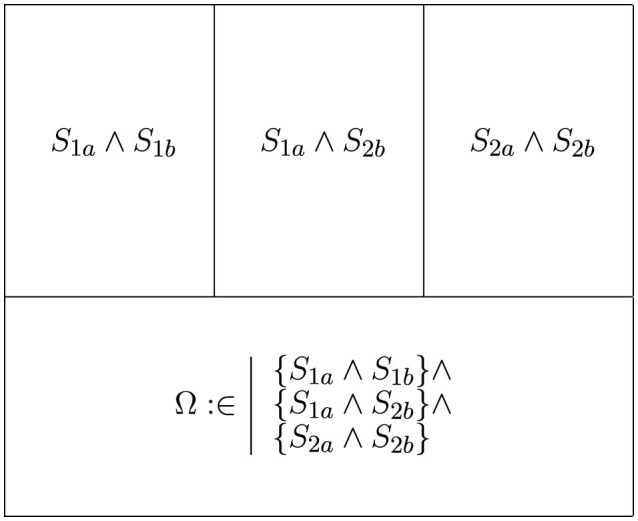
Example solution set with two categories (1, 2) and two topic-specific term sets (a, b). The search string generates three segment types: two discipline-specific segments, one expert overlap segment, and one golden gaps segment.

Creating a cross-disciplinary search requires developing a search string that combines the searches of the involved disciplines. To achieve this, search terms must be structured so that the solution set includes discipline-specific searches and a collaborative search. In our CRIS framework, search terms were categorized, with each category (1, 2, etc.) assigned to topic-specific sets of terms (a, b, etc.). To ensure the search string *S* captures both discipline-specific and collaborative searches, categories are linked by a logical “AND” (see Equation 1 and Figure 2).

**Figure 3:**
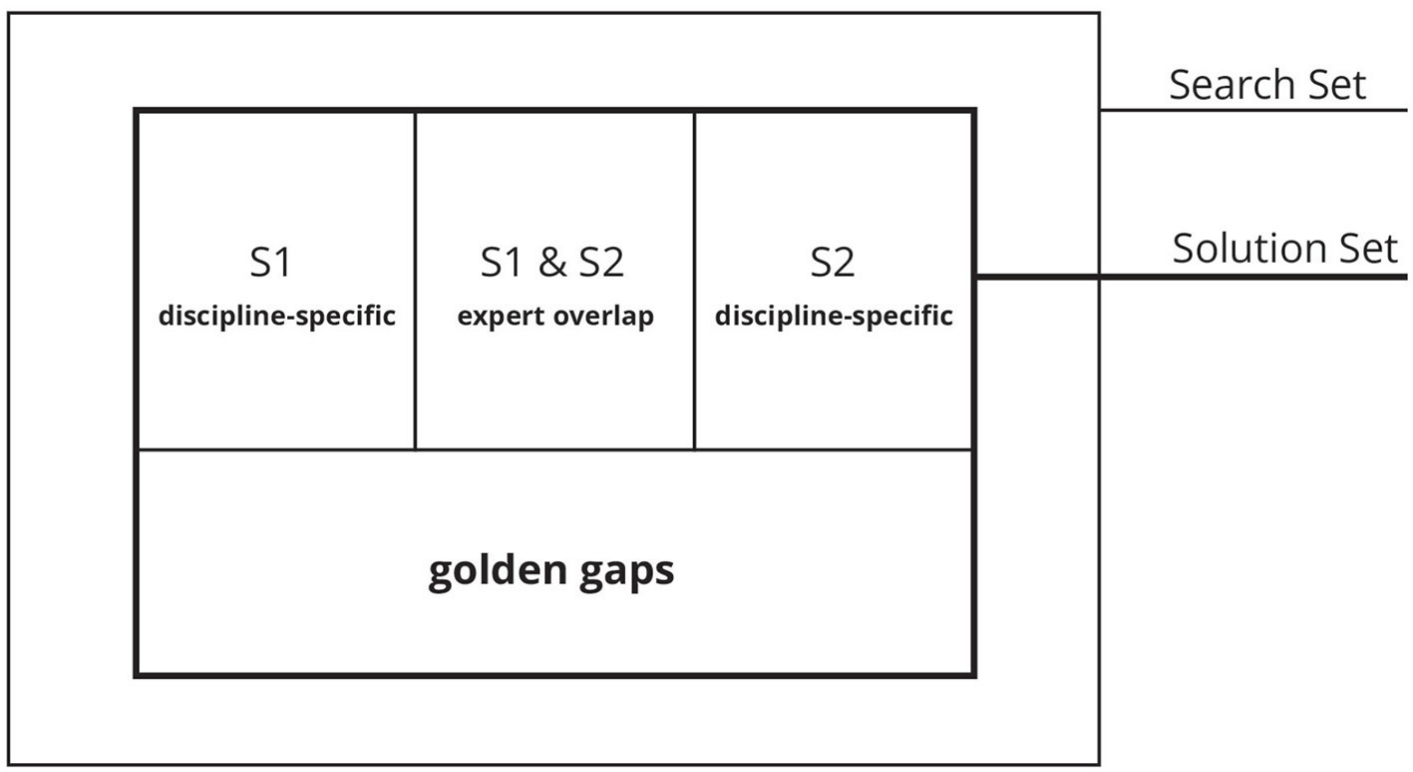
The results encompass the outcomes from database-specific search strings categorized in discipline-specific categories, shown here for two disciplines: S1, S2, the expert overlap of S1 and S2 and golden gaps.

This method results in the identification of three distinct segment types: (1) a segment focused on discipline-specific terms, (2) a segment that captures expert overlap terms, and (3) a segment representing a combination of different discipline-specific categories, as illustrated in Figure 3. Segment 3 is referred to as *golden gaps*.

### 3.2 Walk-through of the cross-disciplinary literature search framework

We apply our CRIS framework through a detailed walk-through of the procedure, using our cross-disciplinary example from the disciplines of HMS and UXG.

#### 3.2.1 Scope determination

In the initial phase, the objective is to establish the scope. This involves clearly understanding how the review will contribute to existing knowledge. It is recommended that well-defined, feasible, and relevant research questions, as well as inclusion and exclusion criteria, be formulated using PICO or its derivatives. Engaging stakeholders and considering existing priority settings can help ensure that the review addresses meaningful issues (32).

In cross-disciplinary systematic reviews, merging discipline-specific and shared scopes presents a distinct challenge, as the scope defined by each discipline can differ substantially. Using CRIS, we emphasize that each corresponding discipline should define its own PICO or derivative. Next, these statements should be synthesized to create a unified PICO or derivative, incorporating discipline-specific subscopes through the use of a shared thesaurus.

In our example, the UXG scope was process-driven, while the HMS scope was outcome-driven. This disparity necessitated a strategy that could incorporate the needs of both disciplines within our search. Therefore, we used the concept of the shared thesaurus and developed a PICO derivative tailored to address the scope of both our disciplines. In the first step, each discipline defined its interpretation of the scope, guided by the PICO structure, and included the options to explore outlined in the Lefebvre et al. (24). Although PICO was highly applicable for HMS, it did not fully capture the scope of UXG (49). To accommodate UXG's process-oriented focus, we introduced an additional element, the *design approach*. The *comparison* component, crucial for HMS, where randomized controlled trials are the gold standard, was excluded from UXG's scope, as it did not contribute to answering the discipline-specific research question. Subsequently, the scopes of each discipline were combined. We developed a mutual understanding of each discipline's unique needs through discussion sessions and adopted an inclusive approach. This led to creating a unified scope that reflects individual disciplinary scopes and a shared scope. In our example, this unified scope encompasses P (population), D (design approach), I (intervention), C (comparison), O (outcomes), and S (study design).

#### 3.2.2 Identification: selecting information sources

The identification phase is centered on creating the solution set. Therefore, it is suggested that appropriate information sources be carefully selected and search strategies planned to ensure a structured and systematic approach to identifying relevant results for a specific research topic. The selection of databases and information sources is crucial in determining the scope of the search set. It is recommended that relevant databases, along with other information sources such as registers and gray literature, be identified and specified if needed to ensure that the review fully represents the scope of the research topic (16).

Rethlefsen et al. (31) emphasizes that different databases and platforms often have distinct focuses, such as disciplinary, specialized, or regional, and they vary in their search capabilities, including platform-specific field codes and the ability to search within certain metadata, such as titles, abstracts, keywords, or full texts. Additionally, databases differ in their use of controlled vocabularies and classification systems; for instance, PubMed uses Medical Subject Headings (MeSH), IEEE relies on the IEEE Thesaurus, and ACM DL employs the 2012 ACM Computing Classification System (CCS), while some databases may not use such specific regulations. These differences among information sources can have a meaningful impact on the results obtained (55–57). It is important to note that the solution set includes entries from different databases, allowing for overlaps between entries from individual databases.

During our literature search, we faced the challenge of selecting databases that met the disciplines' diverse needs while ensuring relevance from each discipline's perspective. To address this, we applied the concept of focus to categorize our cross-disciplinary scope and define the solution set. This categorization was carried out in two main ways: discipline-specific and across disciplines. The selection process involved consulting experts in HMS and UXG, reviewing database descriptions, and considering the databases' controlled vocabulary and classification systems. For example, ACM Digital Library (ACM DL) was selected from UXG due to its focus on information technology, PubMed was chosen from HMS for its emphasis on medical topics, and Web of Science was included from both disciplines because of its interdisciplinary nature.

#### 3.2.3 Identification: planning of search strategies

The pre-process of our procedure is designed to enhance the transparency and reproducibility of planning search strategies and derive database-specific search strings that effectively identify relevant results. The selection of appropriate search terms, along with their specialized term depth and the way they are combined, is crucial to this process. Possible options for conducting search strategies across disciplines include:

- Pure expert term search: in this approach, experts from each discipline define the key terms specific to their field. The search strategy then involves intersecting these expert terms to create a unified search representing all disciplines' combined expertise.
- Expert term search from a single perspective: in this approach, experts from one discipline select search terms. They use specialized terms tailored to their own discipline, reflecting a discipline-specific expert perspective, while more general terms are chosen for the other disciplines, representing an external perspective. This results in a one-sided perspective cross-disciplinary search.

A critical challenge arises in identifying cross-disciplinary results that are neither solely from an expert's perspective nor entirely from the viewpoint of a single discipline. Cross-disciplinary research can take various forms, including multidisciplinary, interdisciplinary, and transdisciplinary approaches, each with different degrees of evolving specialized language. To address this complexity, our cross-disciplinary search must encompass all these aspects, including the specific database focus, to ensure high sensitivity. The CRIS framework tackles this challenge through two steps in the pre-process: creating the foundational search matrix and preparing the database search.

##### 3.2.3.1 Creating the foundational search matrix

We developed the process of creating the *Foundational Search Matrix* (FSM) by applying the concepts of a shared thesaurus, focus, and iterative approach. The FSM is the grounding for developing database-specific search strings within our procedure. The creation starts by transforming the unified scope into categories. Each discipline derives subcategories from these categories based on its unique perspective, which are then integrated across viewpoints. A matrix (see Table 1) is constructed, with one axis representing the subcategories and the other representing the disciplinary perspectives. This structure enables a focus on both internal discipline-specific perspectives of the subcategories and the external viewpoints of different disciplines.

**Table 1:** This is a template for a foundational search matrix for two disciplines.

**Category**	**Subcategory**	**Perspective a**	**Perspective b**
1	Discipline 1	Search term set	Search term set
	Discipline 2	Search term set	Search term set
2	Discipline 1	Search term set	Search term set
	Discipline 2	Search term set	Search term set
…	…	…	…..
	…	…	…..

Each category is divided into subcategories, which are then assigned search term sets from the discipline-specific perspectives.

In our example, each aspect of PDICOS represents a category. To derive subcategories, the outcomes category, for instance, is divided into *UXG outcomes*, which relate to experiences, and *HMS outcomes*, which focus on motor and/or cognitive aspects.

**Figure 4:**
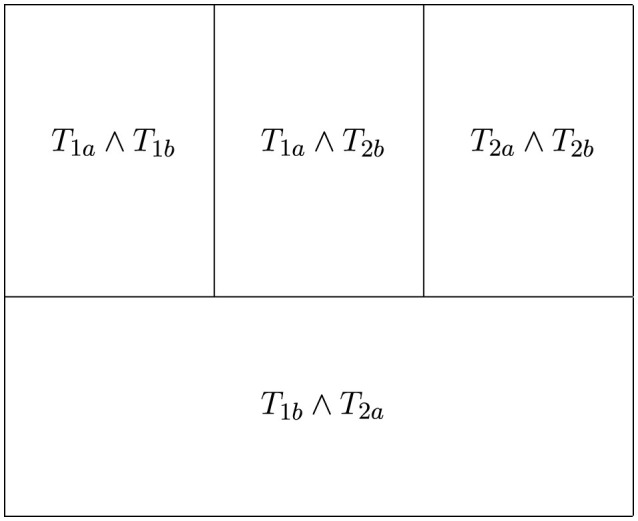
Four segments of a category: two discipline specific, one expert overlap and one gerneralistic.

Within the created FSM, each category can be viewed as comprising four distinct segments based on its subcategories and perspectives, reflecting the specialized term depth, as illustrated in Figure 4. These segments include two discipline-specific term segments (*T*_1*a*_∧*T*_1*b*_, *T*_2*a*_∧*T*_2*b*_), which use the specialized language of each discipline: one expert overlap term segment (*T*_1*a*_∧*T*_2*b*_), where the language fully overlaps, and a general term segment (*T*_1*b*_∧*T*_2*a*_), where there are partial overlaps. This systematic grouping can help assess whether the search terms suit each category.

In our example, involving two disciplines, this process results in four distinct areas. Each discipline then assigns search terms to its subcategories, ensuring that terms from all perspectives are applied to both their own and other disciplines' subcategories. In our example, each subcategory is analyzed from the perspectives of the two disciplines involved: UXG (a) and HMS (b). This careful assignment was crucial, as cross-disciplinary research can involve varying definitions and specialized term depths depending on the content and context. The sets of terms were identified iteratively, leveraging the team's expertise and employing various methods, such as data mining. The complete search matrices are available in Ciemer et al. (29), in the supplementary material (Data Sheet 2 | Search Matrices).

##### 3.2.3.2 Preparing the database search

The key concepts of shared thesaurus, focus, and iterative approach guided the development of the preparing the database search step. This step aims to transform the FSM into concrete database-specific search strings. To begin, the challenge of differing database focuses is addressed by tailoring the FSM to align with each database's specific focus. This involves creating a sub-step integrating insights from the information source phase and incorporating relevant database-specific terms into the term sets.

Based on the resulting database-specific FSMs, an additional sub-step transforms these into search strings. The shared thesaurus guides this sub-step and employs the Boolean Search Query method. Specific rules for constructing the search strings ensure the capture of cross-disciplinary entries across the forms as mentioned earlier from (1) discipline-specific perspectives to (2) expert overlap viewpoints, including (3) golden gaps, which are terms that do not rely on specialized language in at least one subcategory (see Figure 3). These golden gaps become particularly important when an entry uses generalized terms in at least one category. Addressing these forms of cross-disciplinary collaboration ensures that relevant entries, which may not use discipline-specific terminology in one of the categories but are present in the shared thesaurus, are included. Additionally, ensuring that every subcategory is represented in the search is essential. The rules for combining the search terms from the database-specific FSMs are presented in Equation 2. It elaborates that each subcategory search string consists of discipline-specific term sets *T*_*i*_, connected by “OR” logic and categories by “AND” logic. These sets include terms from the disciplines' internal and external perspectives, ensuring that at least one term from *T*_*i, j*_ is included. We developed a search string generator to easily transform the database-specific FSMs into search strings according to our rules. The search strings are available in Ciemer et al. (29), in the supplementary material (Data Sheet 3 | Search Strings).


(2) $$\begin{array}{l} S_{n}=\overset{l}{\underset{i=1}{\wedge }}\overset{p}{\underset{j=1}{\vee }}\left(T_{ij}\right) \end{array}$$

We introduced two additional sub-steps to tailor further and validate the database search strings. Guided by the concepts of iterative approach and focus and drawing on techniques from Phases 1 and 3 of PALETTE (35), we conducted an iterative process to assemble golden bullet sets for each database. The terms in the search strings were carefully refined to maximize relevance. In the subsequent sub-step, the search strings were validated by ensuring they effectively captured the golden bullets.

#### 3.2.4 Identification: performing the database search

The search is performed by applying the database-specific search strings within their respective databases. In our example, we ensured that the filtering options of each database were used as consistently as possible to achieve a uniform and thorough search. To address potential heterogeneity across databases, we recommend employing an alignment script to maintain the uniformity and accuracy of the search results. In our example, we developed a routine to harmonize our search process. Therefore, the metadata from the solution sets were downloaded as BibTeX files, and any duplicates as well as entries that did not meet our inclusion and exclusion criteria—such as systematic reviews or books—were excluded. For example, entries with the word “review” in the title or those marked as books in the metadata were removed.

#### 3.2.5 Screening and inclusion

During the screening phase, it is determined which entries will be included in the review and which are irrelevant to the topic. This process can be done entirely by humans or with automation tools (30).

##### 3.2.5.1 Exclusion of entries via automation tool

The purpose of an automation tool in the screening process is to enhance robustness and transparency in decision-making while simplifying the workload and expediting the review and selection of relevant studies (58, 59). To achieve this purpose in our cross-disciplinary search and ensure a transparent exclusion process, we developed a semi-automated method that applies the concepts of shared thesaurus and iterative approach. This method involves creating sets of terms based on the scope and the inclusion/exclusion criteria, systematically filtering entries according to these sets, and then validating the filtered entries through a specialized sampling process.

Using our method, it is advisable to begin by manually reviewing a selection of the solution set and carefully examining the titles, abstracts, and keywords to gain a clear understanding of the data and identify terms that warrant the exclusion of an article. These exclusion terms are then organized into groups, with related terms added to each group. For instance, our example focused exclusively on older adults. However, we noticed that many entries also included other target groups, such as adolescents and children. To address this, we created a set of child-related terms, including terms like “child,” “toddler,” and “girl.”

Next, we employed a script to systematically apply the term sets as filters across the entire solution set. The script leverages metadata—such as keywords, abstracts, and titles from the extracted BibTeX files—to classify entries. To ensure minimal errors, we developed a specialized process. The filters are applied individually. After each filter was used, the excluded entries were grouped for review. In this group, the terms that led to their exclusion are highlighted, enabling a quick review and decision-making process. If a false negative is detected, the process is iterated by refining the filters and reassessing the previously misclassified entry or marking it as included.

##### 3.2.5.2 Exclusion of entries by human

In the exclusion of entries by humans, titles and abstracts are examined, followed by retrieving the full text of reports deemed potentially relevant (24).

In a cross-disciplinary screening process, excluding entries across disciplines requires that the review team collectively possess the full spectrum of necessary expertise. This is especially crucial for assessing entries specific to particular disciplines, which experts in those fields should evaluate. To address this challenge, we employed the concept of focus. Each reviewer examined every entry in our cross-disciplinary screening, classifying it as relevant, neutral, or excluded. If an entry was deemed relevant by at least one discipline, a discussion was held to resolve any uncertainties. If, after discussion, the entry remained relevant to at least one discipline, it was included in the review, as the goal of the cross-disciplinary literature search is to capture all perspectives on the topic. As a result, data extraction and analysis may require multiple methods or approaches combining different methodologies, such as mixed-methods.

### 3.3 Evaluation of the cross-disciplinary literature search framework

We validated our CRIS framework by comparing it with discipline-specific searches in HMS and UXG, as well as an expert overlap search, using our cross-disciplinary example. Therefore, we calculated the relative sensitivity and assessed the specificity.

**Figure 5:**
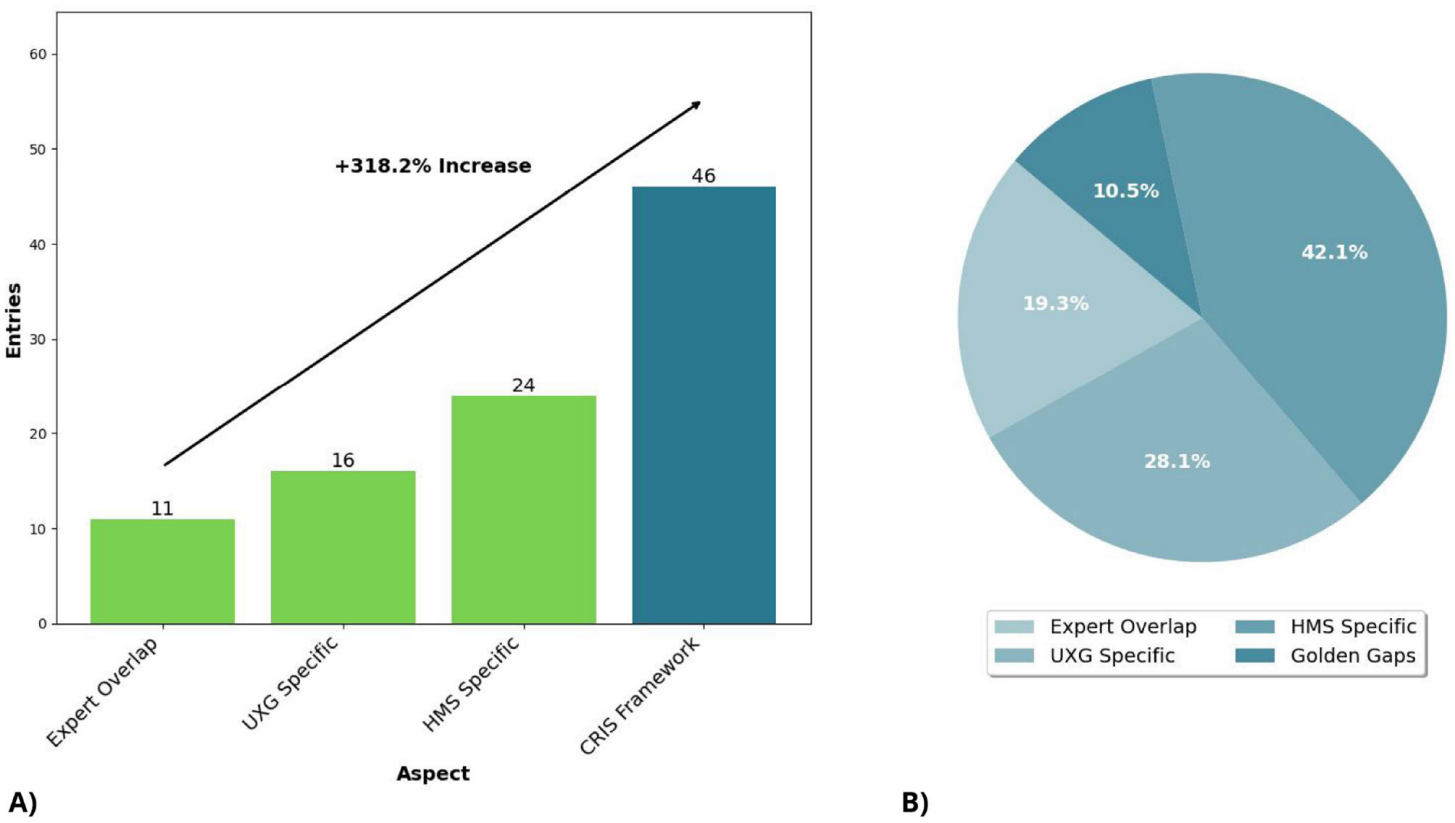
**(A)** Distribution of the number of entries across the categories: expert overlap, discipline-specific (HMS and UXG), and the cross-disciplinary literature search (CRIS) framework. **(B)** Distribution of entries within the CRIS framework.

The cross-disciplinary literature search demonstrated a significant increase in sensitivity, showing a 418.2% rise compared to the expert overlap search (see Figure 5). In the expert overlap search, 11 positive entries were identified, while the UXG-specific search found 16, the HMS-specific search found 24, and the CRIS framework identified 46 positive entries. It is important to note that entries from different information sources were categorized into distinct groups. To calculate this composition, we treated entries appearing in multiple databases as duplicates, resulting in a base of 57 entries. These entries were distributed as follows: 42.1% HMS-specific, 28.1% UXG-specific, 19.3% expert overlap, and 10.5% identified as golden gaps.

**Figure 6:**
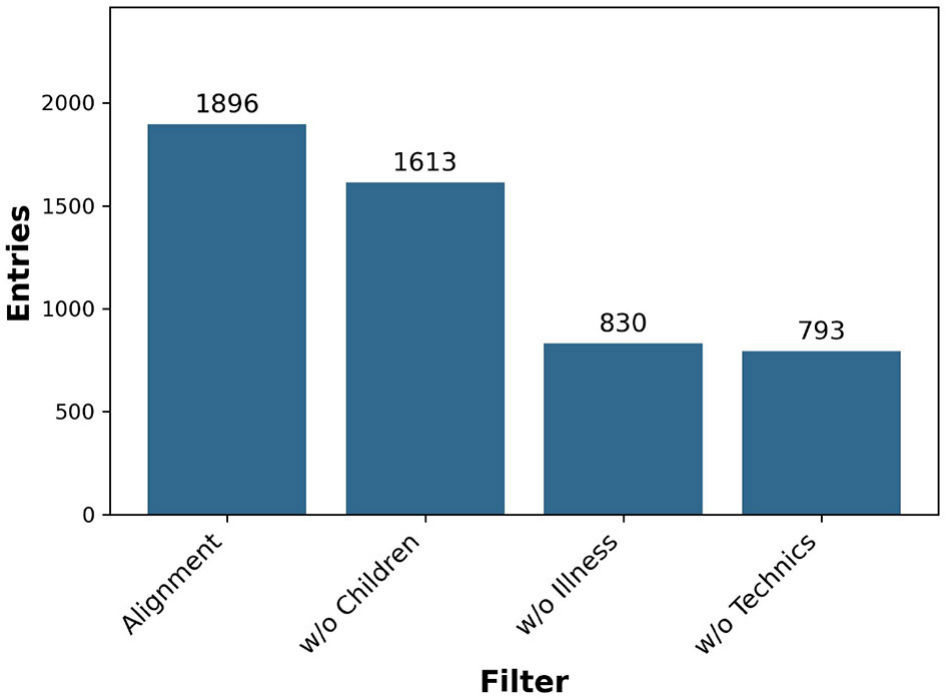
Filtered entries by applying grouped term sets.

The relative specificity cannot be calculated due to the unknown number of false negative entries. However, it is evident that our search results in a decrease in specificity. We quantified the impact of applying the semi-automated grouping method for filtering. Prior to the application of our alignment script, we had 1,896 unfiltered entries. By employing our semi-automated grouping method, as illustrated in Figure 6 we created three distinct term sets as filters. This method enabled us to reduce the number of entries to 793, achieving an overall reduction of 58.2%. This reduction was accomplished through three stages: first, the filter for child-related terms narrowed the entries to 1,613. Next, the filter for disease-related terms further reduced the count to 830 entries. Finally, applying the filter for computer science terms decreased the number to 793 entries.

## 4 Discussion

This article introduces a framework for conducting cross-disciplinary literature searches (CRIS) for open unified questions in a transparent and structured manner designed to address the diverse requirements that emerge from cross-disciplinary collaboration. By adapting the concepts of focus, shared thesaurus, and iterative approach, we aimed to create a robust and sensitive framework that integrates seamlessly with the PRISMA guidelines and aligns with standard recommendations (16, 24, 30, 32, 33).

Given the absence of established guidelines specifically for cross-disciplinary searches, our framework addresses a critical gap in the literature. The increasing importance of cross-disciplinary collaboration in research necessitates the development of search strategies that can accommodate the broader scope and diverse linguistic nuances inherent in such studies (1, 2, 6). Our CRIS framework is particularly relevant for ensuring that no aspect of a cross-disciplinary topic is overlooked while managing the evolving nature of specialized language, integrating the varied perspectives across different fields, and harmonizing the distinct emphases of multiple disciplines within a single systematic review. To our knowledge, no existing search framework has focused on these challenges, underscoring the essential nature of our proposed framework.

One of the key benefits of a cross-disciplinary search is its ability to incorporate a broader range of studies into a systematic review. Our findings demonstrate that by employing steps such as creating FSMs, the subject matter is explored from the intrinsic expert perspective and a more generalized external perspective. Our framework identified more comprehensive results, even when less specialized terminology was used, resulting in ~10% of the findings by identifying golden gaps. Compared to a expert overlap search, the overall increase in identified studies was nearly fourfold, as evidenced by our sensitivity analysis. Including a more significant number of studies enhances the overall quality of the review by providing a more accurate representation of the current state of research. Our framework effectively accommodates a wide array of cross-disciplinary collaboration forms.

Furthermore, our CRIS framework has shown the potential to enhance the robustness of literature searches. This conclusion is supported by the observation that identical studies were identified using different search strings across various databases. The comprehensive nature of our search string ensures that relevant entries are captured even when discipline-specific strings might miss them in specific databases. This adaptability allows our search strategy to meet different databases' varying demands effectively.

However, including all relevant research outputs leads to a reduction in specificity. While an increase in the number of results can enhance the scope of the review, it can also result in the identification of more irrelevant studies, thereby increasing the workload. Accelerating the screening process through automation tools is crucial to mitigate this. Our method suggests creating word groups and applying filters sequentially, which, combined with topic-specific filtering and highlighting of key terms, can reduce the effort required while maintaining the quality of the screening process. Our findings indicate that our approach results in lower specificity than expert overlap searches, highlighting areas where further optimization is necessary.

Using CRIS, included entries may require more complex analyses due to variations in approaches, methodologies, standards, or epidemiological differences when addressing the research questions. This creates new opportunities and may necessitate analyses for specific groups or mixed-methods approaches [e.g., using a convergent integrated approach or a convergent segregated approach (17)]. Additionally, it may lead to the development of new analytical methods that refine the understanding of a topic and provide deeper insights into the cross-disciplinary research subject.

## 5 Limitations

Several factors must be considered in addressing the limitations of our cross-disciplinary literature framework. First, using one cross-disciplinary example, our framework has only been applied and tested in a single instance. This limited application raises concerns about the generalizability of our findings. Additionally, our comparisons were primarily made with an expert overlap search, for which there are currently no specific guidelines. This lack of established standards may weaken the strength of our conclusions, as it is challenging to benchmark our results effectively.

Furthermore, these limitations restrict the broader applicability of our framework. Although we demonstrated the effectiveness of our approach within the context of our example, this does not guarantee its applicability to other systematic reviews, which may have different scopes, objectives, and constraints. Also, the framework cannot be used if disciplines cannot find a unified scope. Our framework was also developed and tested exclusively within the confines of our specific disciplinary boundaries. Other fields may have unique requirements and follow different guidelines for conducting systematic reviews, meaning our framework may need adaptation to be effective in other cross-disciplinary contexts.

Finally, a notable challenge we identified is efficiently managing the large volume of search results that our high-sensitivity approach can yield. While our strategy aims for comprehensive coverage, this often results in an extensive solution set, which can be difficult to handle effectively.

## 6 Conclusion

In this article, we have demonstrated and validated a framework for conducting cross-disciplinary literature searches (CRIS) within systematic reviews. This framework is intended to guide researchers undertaking cross-disciplinary literature searches, addressing the unique requirements of cross-disciplinary research. By integrating the concepts of thesaurus, focus, and an iterative approach, we developed a procedure, including a pre-process, that aligns with PRISMA guidelines and established recommendations. The framework was validated through three separate searches conducted in collaboration with the disciplines of User Experience and Game Design and Human Movement Science. Our findings indicate that this framework offers a fourfold increase in sensitivity compared to an expert overlap search. Additionally, by considering the distinct focuses and requirements of different databases and topics, the robustness of the search process was enhanced. Future work should seek to validate this framework in other contexts and explore ways to increase its specificity.

## Data Availability

The original contributions presented in the study are included in the article/supplementary material, further inquiries can be directed to the corresponding author.
